# Gender-Specific Outcomes in TAVI with Self-Expandable Valves: Insights from a Large Real-World Registry

**DOI:** 10.3390/jcm14093144

**Published:** 2025-05-01

**Authors:** Alessandro Sticchi, Dario Grassini, Francesco Gallo, Stefano Benenati, Won-Keun Kim, Arif A. Khokhar, Tobias Zeus, Stefan Toggweiler, Roberto Galea, Federico De Marco, Antonio Mangieri, Damiano Regazzoli, Bernhard Reimers, Luis Nombela-Franco, Marco Barbanti, Ander Regueiro, Tommaso Piva, Josep Rodés-Cabau, Italo Porto, Antonio Colombo, Francesco Giannini

**Affiliations:** 1 Dipartimento di Patologia Chirurgica, Medica, Molecolare e dell’Area Critica, Lungarno Antonio Pacinotti, University of Pisa, 43, 56126 Pisa, Italy; 2 Department of Biomedical Sciences, Humanitas University, 20072 Milan, Italycolombo.emogvm@gmail.com (A.C.); 3 Cardio Center, IRCCS Humanitas Research Hospital, 20089 Milan, Italy; 4 Interventional Cardiology, Department of Cardio-Thoracic and Vascular Sciences, Ospedale dell’Angelo, AULSS3 Serenissima, Mestre, 30174 Venezia, Italy; galfra87@gmail.com; 5 Dipartimento di Medicina Interna e Specialità Mediche (DIMI), University of Genoa, 16126 Genoa, Italy; 6 Department of Cardiology, Kerckhoff Heart Center, 61231 Bad Nauheim, Germany; 7 Cardiology Service, Imperial College Healthcare NHS Trust, London W12 0HS, UK; 8 Division of Cardiology, Pulmonology and Vascular Medicine, Heinrich Heine University, 40225 Duesseldorf, Germany; 9 Department of Cardiology, Cantonal Hospital Lucern, 6000 Luzern, Switzerland; 10 Department of Cardiology, Inselspital, Bern University Hospital, University of Bern, 3010 Bern, Switzerland; 11 Centro Cardiologico Monzino IRCCS, 20100 Milan, Italy; 12 Cardiovascular Institute, Hospital Clínico San Carlos, Institute of the Hospital Clínico San Carlos, 28040 Madrid, Spain; 13 Faculty of Medicine and Surgery, Università degli Studi di Enna “Kore”, 94100 Enna, Italy; 14 Cardiovascular Institute, Hospital Clinic, Institut D’investigacions Biomèdiques August Pi I Sunyer (IDIBAPS), 08036 Barcelona, Spain; 15 Interventional Cardiology, Ospedali Riuniti Di Ancona, 60126 Ancona, Italy; 16 Quebec Heart and Lung Institute, Laval University, Quebec City, QC G1V 4G5, Canada; 17 Interventional Cardiology Unit, IRCCS Ospedale Galeazzi Sant’Ambrogio, 20157 Milan, Italy

**Keywords:** TAVI, female, gender differences, self-expandable valves, clinical outcomes, propensity score matching

## Abstract

**Background/Objectives**: Aortic stenosis (AS) is the most prevalent valvular heart disease in developed countries and imposes an increasing burden on aging populations. Although transcatheter aortic valve implantation (TAVI) has transformed the treatment of severe AS, current guidelines do not differentiate management based on gender. This study aimed to investigate gender-based differences in procedural complications and one-year clinical outcomes in patients treated with next-generation self-expandable TAVI devices. **Methods**: This retrospective, multicenter international registry included 3862 consecutive patients who received either the ACURATE neo or Evolut R/Pro valve. Patients were stratified by gender; propensity score matching (PSM) adjusted for baseline differences. The primary endpoint was a composite of all-cause mortality or stroke at one year. Secondary endpoints included major vascular complications, major or life-threatening bleeding and acute kidney injury (AKI). **Results**: Of 3353 patients included (64.5% female), women were older (82.3 ± 5.6 vs. 81.1 ± 6.2 years, *p* < 0.001) and had higher STS scores (5.2 ± 3.9 vs. 4.5 ± 3.4%, *p* < 0.001). In the unmatched population, major vascular complications occurred in 7.7% of females versus 4.1% of males (*p* < 0.001), life-threatening bleeding in 2.8% vs. 1.4% (*p* = 0.016) and AKI in 8.5% vs. 5.7% (*p* = 0.009). After PSM, the primary endpoint was more frequent in females (9.4% vs. 6.0%, *p* = 0.014), largely driven by stroke (2.8% vs. 1.2%, *p* = 0.024), while overall mortality was similar (11.3% vs. 9.5%, *p* = 0.264). **Conclusions**: Despite comparable long-term survival, female patients undergoing TAVI with self-expandable valves experience higher rates of procedural complications, notably stroke and major vascular events. These findings underscore the need for tailored procedural strategies to improve outcomes in female patients.

## 1. Introduction

Aortic stenosis (AS) is the most common form of valvular heart disease in developed countries, particularly affecting the elderly population [1,2]. Without valve replacement, symptomatic severe AS is associated with high mortality [3]. In recent years, transcatheter aortic valve implantation (TAVI) has transformed the treatment of AS, expanding from high-risk patients to those at intermediate and lower surgical risk [4,5,6].

Despite the widespread adoption of TAVI, gender-based differences in clinical outcomes remain underexplored. Women undergoing TAVI often present distinct clinical and anatomical characteristics compared to men, including older age and smaller vascular dimensions, which may influence procedural risks and outcomes [4,7]. However, current clinical guidelines do not differentiate management based on gender, and existing evidence regarding gender-specific outcomes remains conflicting [8,9,10].

Understanding these differences is crucial for optimizing procedural planning and improving outcomes. Recent studies have suggested higher rates of procedural complications such as major vascular events, bleeding and stroke in female patients, but data remain limited, particularly for next-generation self-expandable valves [10,11,12,13,14,15,16,17,18].

This study aims to address this gap by evaluating gender-based differences in procedural complications and one-year clinical outcomes among patients treated with ACURATE neo or Evolut R/Pro valves. Using a large real-world registry and propensity score matching, we sought to isolate the effect of gender while adjusting for baseline characteristics, providing new insights to guide gender-specific strategies in TAVI.

## 2. Materials and Methods

### 2.1. Study Design and Population

This retrospective, multicenter, international registry analysis evaluated patients who underwent TAVI using two next-generation self-expandable bioprostheses, ACURATE neo and Evolut R/Pro valves. Data were collected from consecutive patients at European high-volume TAVI centers. The aim was to assess gender-based differences in clinical characteristics, procedural outcomes and one-year clinical endpoints. Initially, 3862 consecutive patients were included in the registry. For the purposes of this analysis, patients were divided into two cohorts based on gender (male and female). Exclusion criteria were limited to patients with incomplete follow-up data or missing key procedural or outcome parameters.

### 2.2. Propensity Score Matching

To account for differences in baseline characteristics and pre-procedural risk factors, a PSM analysis was performed. Propensity scores were generated using logistic regression that incorporated all relevant baseline covariates, including age, body mass index (BMI), comorbid conditions (e.g., diabetes, chronic obstructive pulmonary disease (COPD), peripheral arterial disease [PAD] and prior cardiac surgery) and echocardiographic parameters (e.g., valve area and left ventricular ejection fraction). A 1:1 matching without replacement was applied to pair male and female patients, ensuring that matched pairs had similar baseline characteristics and thereby enabling a more accurate comparison of outcomes between the two groups.

### 2.3. Study Endpoints

The primary endpoint was a composite of all-cause mortality or stroke (disabling and non-disabling) at one year following TAVI. Secondary endpoints included the individual components of the primary endpoint (all-cause mortality and stroke) as well as other clinically significant procedural complications, including major vascular complications, major bleeding, life-threatening bleeding, cardiac tamponade, AKI, the need for blood transfusion, new permanent pacemaker implantation and hospital readmission for heart failure (HF).

### 2.4. Data Collection

All clinical, echocardiographic, procedural and outcome data were prospectively recorded at each participating center using a standardized format [11]. Baseline characteristics included demographic variables (e.g., age and sex), cardiovascular risk factors (e.g., hypertension, diabetes, dyslipidemia and smoking) and detailed procedural data (e.g., valve type, valve size, use of predilatation and post-dilatation and vascular access). Imaging data, including echocardiographic and computed tomography parameters, were assessed by local heart teams. Post-procedural clinical outcomes, including adverse events, were defined according to the Valve Academic Research Consortium (VARC) criteria [12]. Major vascular complications were defined as those resulting in death, major bleeding, visceral ischemia, or necessitating surgical intervention. Acute kidney injury was defined according to the Kidney Disease: Improving Global Outcomes (KDIGO) classification [13]. All endpoints were adjudicated by an independent clinical events committee.

### 2.5. Statistical Analysis

Categorical variables are expressed as counts and percentages, while continuous variables are presented as means ± standard deviations (SD) or medians with interquartile ranges (IQRs), as appropriate. Comparisons between male and female patients were performed using the chi-square test or Fisher’s exact test for categorical variables and the Student’s *t*-test or Mann–Whitney U test for continuous variables. To account for potential confounders in the unmatched cohort, multivariable logistic regression was performed to identify independent predictors of the primary composite outcome; odds ratios (OR) with 95% confidence intervals (CI) were calculated for each variable. In the PSM cohort, outcomes were compared between men and women using McNemar’s test for categorical variables and paired *t*-tests for continuous variables. Statistical significance was defined as a two-tailed *p*-value of less than 0.05. All statistical analyses were performed using SPSS v.22 (IBM^®^ SPSS^®^ Statistics, 1 New Orchard Road Armonk, New York, NY, USA).

### 2.6. Ethical Considerations

The study protocol conformed to the ethical guidelines of the 1975 Declaration of Helsinki [14], and approval was obtained from the institutional review boards of all participating centers. Given the retrospective and observational nature of the study, the requirement for informed consent was waived at each institution; however, all patient data were anonymized to protect privacy.

## 3. Results

### 3.1. Baseline Characteristics (Pre-PSM)

A total of 3353 patients who underwent TAVI with either the ACURATE neo or Evolut R/Pro valves were included in the analysis. Of these, 2162 (64.5%) were female and 1191 (35.5%) were male. Female patients were, on average, older than male patients (82.3 ± 5.6 years vs. 81.1 ± 6.2 years, *p* < 0.001), had a higher BMI (27.7 ± 5.7 kg/m^2^ vs. 27.2 ± 4.5 kg/m^2^, *p* = 0.023) and exhibited a lower prevalence of dyslipidemia (50.2% vs. 54.7%, *p* = 0.037). Women also had lower rates of diabetes (26.8% vs. 33.7%, *p* < 0.001), smoking history (10.0% vs. 24.3%, *p* < 0.001), COPD (17.4% vs. 21.9%, *p* = 0.002) and prior cardiac surgery (8.6% vs. 18.8%, *p* < 0.001). The mean STS score was significantly higher in women (5.2 ± 3.9% vs. 4.5 ± 3.4%, *p* < 0.001). Imaging data revealed that women had smaller aortic valve areas (0.7 ± 0.2 cm^2^ vs. 0.9 ± 4.1 cm^2^, *p* = 0.037), higher mean valve gradients (45.4 ± 17.1 mmHg vs. 42.7 ± 14.7 mmHg, *p* < 0.001) and smaller annular perimeters (56.8 ± 23.0 mm vs. 62.0 ± 23.8 mm, *p* < 0.001). Full details of the baseline characteristics are presented in Table 1 and Table 2.

### 3.2. Procedural Characteristics (Pre-PSM)

Procedural characteristics were stratified by gender. Transfemoral access was used more frequently in male patients (63.5% vs. 53.9%, *p* < 0.001), whereas women more often underwent procedures with the Evolut R valve (41.5% vs. 36.5%, *p* = 0.006). Post-dilatation was required more commonly in male patients (35.2% vs. 30.0%, *p* = 0.002), and the mean balloon size for post-dilatation was larger in males (16.3 ± 11.3 mm vs. 13.6 ± 11.2 mm, *p* < 0.001). Full procedural details are available in Table 3.

### 3.3. Procedural Complications and Secondary Outcomes (Pre-PSM)

The incidence of procedural complications varied significantly between genders. Female patients experienced higher rates of cardiac tamponade (1.5% vs. 0.4%, *p* = 0.008), major vascular complications (7.7% vs. 4.1%, *p* < 0.001), life-threatening bleeding (2.8% vs. 1.4%, *p* = 0.016) and major bleeding (5.1% vs. 2.9%, *p* = 0.004). Additionally, the need for blood transfusion was significantly higher in women (8.9% vs. 4.6%, *p* < 0.001), as was the incidence of acute kidney injury (AKI) (8.5% vs. 5.7%, *p* = 0.009). There was no significant difference in the incidence of annular rupture or aortic regurgitation between the groups. Detailed outcomes are presented in Table 4.

### 3.4. Primary and Secondary Outcomes (Pre-PSM)

In the unmatched population, the primary endpoint, a composite of all-cause death or stroke at one year, occurred at similar rates in women and men (7.9% vs. 6.9%, *p* = 0.337). Likewise, there was no significant difference in all-cause mortality (5.9% vs. 5.6%, *p* = 0.786) or stroke rates (2.5% vs. 1.8%, *p* = 0.243). However, women exhibited higher in-hospital mortality compared to men (1.6% vs. 0.6%, *p* = 0.016). Full clinical outcome data are summarized in Table 5.

### 3.5. Baseline Characteristics (Post-PSM)

After propensity score matching, 844 female patients were matched with 844 male patients, resulting in two cohorts with similar baseline characteristics. There were no significant differences in age, BMI, or the prevalence of cardiovascular risk factors—including diabetes, dyslipidemia and COPD. The matched cohorts also exhibited similar echocardiographic parameters, such as mean pressure gradients, ejection fraction and annular dimensions. Baseline characteristics and pre-procedural imaging parameters after PSM are detailed in Table 6 and Table 7, respectively.

### 3.6. Procedural Characteristics (Post-PSM)

In the propensity-matched cohort, procedural characteristics remained balanced between the groups. The use of transfemoral access, predilatation, and post-dilatation was similar between men and women, although women more frequently received the Evolut R valve (44.8% vs. 35.0%, *p* < 0.001). The balloon size used for post-dilatation and the contrast volume administered were not significantly different between genders. Full procedural characteristics are provided in Table 8.

### 3.7. Procedural Complications and Secondary Outcomes (Post-PSM)

Despite matching for baseline characteristics, gender differences in procedural complications persisted. Women continued to experience higher rates of major vascular complications (6.8% vs. 4.1%, *p* = 0.024), the need for blood transfusion (9.1% vs. 4.6%, *p* < 0.001) and AKI (8.7% vs. 5.6%, *p* = 0.029). There was no significant difference in the rates of cardiac tamponade or emergency surgery between men and women. These findings are summarized in Table 9.

### 3.8. Primary and Secondary Outcomes (Post-PSM)

In the matched cohort, the primary endpoint of all-cause death or stroke at one year was significantly higher in women (9.4% vs. 6.0%, *p* = 0.014). Stroke was more prevalent among female patients (2.8% vs. 1.2%, *p* = 0.024), although there was no significant difference in all-cause mortality (11.3% for women vs. 9.5% for men, *p* = 0.264). Additionally, rates of major bleeding and life-threatening bleeding were higher in women, though these did not reach statistical significance. Full clinical outcome data after PSM are presented in Table 10.

## 4. Discussion

This study represents one of the largest real-world analyses to date examining gender-based differences in patients undergoing TAVI with next-generation self-expandable bioprostheses. The findings from both the unmatched and PSM cohorts provide significant insights into how gender influences clinical outcomes and procedural characteristics, even after adjusting for baseline disparities.

### 4.1. Baseline Characteristics

Pre-PSM, our cohort revealed distinct differences between men and women, with female patients generally being older, having a higher BMI and presenting with lower rates of cardiovascular risk factors such as dyslipidemia, diabetes and smoking. These gender differences are consistent with previous studies, which have noted that women undergoing TAVI tend to present with fewer traditional cardiovascular risk factors but with more advanced age and greater frailty [10,15,16,17]. After PSM, the baseline characteristics were balanced, allowing for a more accurate assessment of gender-specific outcomes.

### 4.2. Procedural Characteristics and Complications

The higher frequency of transfemoral access and post-dilatation in male patients pre-PSM likely reflects anatomical differences, including larger body surface area and annular dimensions in men, as indicated by pre-procedural imaging parameters. After PSM, baseline profiles were balanced except for kidney function; female patients still exhibited lower eGFR values compared to males. This residual difference may have influenced post-procedural AKI rates and is a relevant limitation that should be considered when interpreting our findings. In fact, although the procedural differences were mitigated after PSM, women continued to experience higher rates of procedural complications, including major vascular events, the need for blood transfusion and AKI. Notably, even after matching, women exhibited significantly higher rates of AKI and major vascular complications, corroborating existing literature and underscoring the vulnerability of female patients to specific procedural risks [16,17,18]. These findings reinforce the need for a sex-specific procedural strategy, including careful vascular access planning (adoption of lower-profile delivery systems and meticulous closure techniques) and low-contrast strategy, particularly in female patients.

### 4.3. Clinical Outcomes

The primary composite outcome of all-cause death or stroke at one year was significantly higher in women post-PSM, largely driven by an increased incidence of stroke. Interestingly, mortality rates between men and women remained similar both before and after PSM. The higher stroke risk in women is consistent with other studies and may reflect anatomical and physiological differences that predispose women to cerebrovascular events during TAVI [16,17,18]. These findings are clinically significant, as they suggest that while female patients are not at a higher risk for mortality, they are more likely to experience stroke, which can profoundly affect post-procedural quality of life and long-term recovery. Greater attention to embolic protection strategies and procedural optimization may be warranted for female patients, particularly those with additional stroke risk factors [19].

### 4.4. Vascular and Bleeding Complications

Women consistently had higher rates of major vascular complications and bleeding, even after adjusting for baseline differences. These observations align with existing data indicating that smaller vascular dimensions and greater vascular fragility in women contribute to an elevated risk of vascular injury during TAVI [20]. These results underscore the importance of tailored vascular strategies, including pre-procedural imaging, minimizing sheath-to-artery ratios and careful device selection to mitigate bleeding and vascular risks in female patients. Despite advances in valve technology and procedural techniques aimed at minimizing these complications, the higher rates of bleeding and transfusion in women highlight the need for further refinement of TAVI strategies in this patient population.

### 4.5. Acute Kidney Injury (AKI)

The higher incidence of AKI among women, observed both before and after propensity score matching, is a significant finding with important clinical ramifications. Although major baseline characteristics were balanced after matching, women still exhibited significantly lower eGFR values compared to men. This residual difference suggests that female patients may have entered the procedure with a higher degree of renal vulnerability, potentially predisposing them to post-procedural kidney injury. Moreover, the interplay between vascular complications, bleeding events and hemodynamic instability, all of which were more frequent among women, may have further contributed to renal insult in this population.

These findings highlight the need for proactive renal protection strategies in female patients undergoing TAVI. Measures such as minimizing contrast agent use, applying strict peri-procedural hydration protocols, avoiding nephrotoxic medications when possible and early identification of renal function deterioration should be prioritized. Moreover, individualized risk stratification for AKI should be integrated into the pre-procedural planning, especially in women with borderline renal function, to further optimize outcomes [21].

### 4.6. Clinical Implications

The persistent gender differences in procedural complications and outcomes after PSM underscore the need for gender-specific strategies in TAVI. For female patients, special attention should be given to minimizing vascular and bleeding complications, as well as addressing the elevated risks of stroke and AKI. These findings suggest that tailored procedural planning, including the use of smaller vascular access devices, careful hemodynamic management and stroke-prevention strategies during the peri-procedural period, may benefit female patients. Moreover, the comparable long-term survival benefits of TAVI between men and women indicate that, despite higher procedural risks, women should not be denied access to TAVI.

### 4.7. Study Strengths and Limitations

The strengths of this study include its large sample size and the use of propensity score matching to adjust for baseline differences between men and women. This approach allowed for a more accurate comparison of outcomes across genders and provided robust real-world data that complement findings from more selective clinical trials. Additionally, focusing on next-generation self-expandable valves reflects contemporary TAVI practice, making these findings highly relevant to current clinical decision-making. However, the retrospective nature of the registry introduces inherent biases, and despite PSM, unmeasured confounders may still influence the outcomes. Moreover, the study was unable to fully explore the pathophysiological mechanisms underlying the observed gender differences—particularly the higher stroke and AKI rates in women. Future studies incorporating detailed anatomical and hemodynamic assessments could help elucidate these mechanisms further.

## 5. Conclusions

In conclusion, this large real-world TAVI registry highlights significant gender differences in procedural complications and clinical outcomes. Female patients undergoing TAVI remain at higher risk for stroke, major vascular complications, bleeding and AKI, even after adjusting for baseline risk factors. These findings call for a more tailored approach to TAVI in women, with strategies aimed at minimizing procedural risks and improving post-procedural outcomes. Further research is needed to explore the underlying mechanisms driving these gender disparities and to develop targeted interventions that enhance the safety and efficacy of TAVI for all patients, regardless of gender.

## Figures and Tables

**Table 1:** Baseline characteristics of the study population (pre-PSM).

Characteristic	Overall (n = 3353)	Female (n = 2162)	Male (n = 1191)	*p*-Value
Age, years (mean ± SD)	81.9 ± 5.8	82.3 ± 5.6	81.1 ± 6.2	<0.001
Male, n (%)	1191 (35.5)	0 (0.0)	1191 (100.0)	<0.001
Height, cm (mean ± SD)	163.6 ± 8.8	160.0 ± 7.0	169.9 ± 8.0	<0.001
Weight, kg (mean ± SD)	72.5 ± 15.3	69.8 ± 15.3	77.3 ± 14.1	<0.001
Body Surface Area, m^2^ (mean ± SD)	1.8 ± 0.2	1.7 ± 0.2	1.9 ± 0.2	<0.001
Body Mass Index, kg/m^2^ (mean ± SD)	27.5 ± 5.4	27.7 ± 5.7	27.2 ± 4.5	0.023
Hypertension, n (%)	2926 (87.4)	1891 (87.6)	1035 (87.0)	0.693
Dyslipidemia, n (%)	1294 (51.8)	804 (50.2)	490 (54.7)	0.037
Diabetes, n (%)	979 (29.2)	578 (26.8)	401 (33.7)	<0.001
Insulin Therapy, n (%)	322 (11.6)	181 (10.0)	141 (14.5)	0.001
History of Smoking, n (%)	281 (15.0)	122 (10.0)	159 (24.3)	<0.001
Chronic Obstructive Pulmonary Disease, n (%)	635 (19.0)	375 (17.4)	260 (21.9)	0.002
Pacemaker/ICD, n (%)	373 (11.1)	207 (9.6)	166 (14.0)	<0.001
Atrial Fibrillation, n (%)	1084 (32.5)	709 (32.9)	375 (31.6)	0.441
Previous Cardiac Surgery, n (%)	408 (12.2)	185 (8.6)	223 (18.8)	<0.001
Previous Myocardial Infarction, n (%)	611 (18.3)	328 (15.2)	283 (23.8)	<0.001
Previous PCI, n (%)	934 (27.9)	496 (23.0)	438 (36.8)	<0.001
Baseline Creatinine, mg/dL (mean ± SD)	1.2 ± 0.7	1.1 ± 0.7	1.3 ± 0.8	<0.001
eGFR, mL/min/1.73 m^2^ (mean ± SD)	53.8 ± 24.1	51.7 ± 23.2	57.7 ± 25.1	<0.001
Chronic Kidney Disease, n (%)	2161 (64.4)	1477 (68.3)	684 (57.4)	<0.001
Dialysis, n (%)	70 (2.3)	35 (1.8)	35 (3.3)	0.010
Peripheral Arterial Disease, n (%)	447 (13.4)	250 (11.6)	197 (16.6)	<0.001
Previous Stroke, n (%)	373 (11.1)	236 (10.9)	137 (11.5)	0.643
NYHA Class III/IV, n (%)	2304 (68.9)	1531 (71.0)	773 (65.0)	<0.001
STS Score (mean ± SD)	5.0 ± 3.7	5.2 ± 3.9	4.5 ± 3.4	<0.001

Abbreviations: BSA = body surface area; BMI = body mass index; COPD = chronic obstructive pulmonary disease; ICD = implantable cardioverter defibrillator; PCI = percutaneous coronary intervention; eGFR = estimated glomerular filtration rate; NYHA = New York Heart Association; STS = Society of Thoracic Surgeons.

**Table 2:** Pre-procedural imaging parameters stratified by gender in the study population (pre-PSM).

Parameter	Overall (n = 3353)	Female (n = 2162)	Male (n = 1191)	*p*-Value
Mean Pressure Gradient, mmHg (mean ± SD)	44.4 ± 16.4	45.4 ± 17.1	42.7 ± 14.7	<0.001
Aortic Valve Area, cm^2^ (mean ± SD)	0.8 ± 2.4	0.7 ± 0.2	0.9 ± 4.1	0.037
Moderate/Severe Aortic Insufficiency, n (%)	303 (18.7)	184 (18.2)	119 (19.5)	0.552
Ejection Fraction, % (mean ± SD)	57.5 ± 10.7	58.5 ± 9.9	55.6 ± 11.8	<0.001
Annulus Area, cm^2^ (mean ± SD)	10.0 ± 11.3	9.7 ± 8.7	10.5 ± 14.9	0.057
Annulus Perimeter, mm (mean ± SD)	58.7 ± 23.4	56.8 ± 23.0	62.0 ± 23.8	<0.001
Aortic Angle > 49°, n (%)	1548 (46.2)	1015 (46.9)	533 (44.8)	0.236
Porcelain Aorta, n (%)	372 (11.1)	247 (11.5)	125 (10.6)	0.464
Moderate/Severe Aortic Valve Calcification, n (%)	1415 (73.5)	882 (71.0)	533 (78.3)	0.001
LVOT Calcification, n (%)	556 (28.9)	358 (28.8)	198 (29.1)	0.924

Abbreviations: LVOT = left ventricular outflow tract.

**Table 3:** Procedural characteristics of the study population (pre-PSM).

Parameter	Overall (n = 3353)	Female (n = 2162)	Male (n = 1191)	*p*-Value
Transfemoral Access, n (%)	1279 (57.4)	767 (53.9)	512 (63.5)	<0.001
Predilatation, n (%)	1792 (53.5)	1132 (52.5)	660 (55.4)	0.108
Evolut R Valve, n (%)	1332 (39.7)	897 (41.5)	435 (36.5)	0.006
Evolut PRO Valve, n (%)	370 (11.0)	218 (10.1)	152 (12.8)	0.021
Evolut R/PRO Valve Combined, n (%)	1702 (50.8)	1115 (51.6)	587 (49.3)	0.218
ACURATE neo Valve, n (%)	1651 (49.2)	1047 (48.4)	604 (50.7)	0.218
Valve Size Categories, n (%)				<0.001
Size 23	153 (4.6)	119 (5.5)	34 (2.9)	
Size 25	14 (0.4)	11 (0.5)	3 (0.3)	
Size 26	645 (19.3)	562 (26.0)	83 (7.0)	
Size 27	8 (0.2)	5 (0.2)	3 (0.3)	
Size 29	894 (26.7)	435 (20.2)	459 (38.6)	
Size 31	8 (0.2)	0 (0.0)	8 (0.7)	
Valve Size Grouped by Category, n (%)				<0.001
Large (L)	552 (16.5)	173 (8.0)	379 (31.8)	
Medium (M)	682 (20.4)	498 (23.1)	184 (15.5)	
Small (S)	392 (11.7)	355 (16.5)	37 (3.1)	
Post-dilatation, n (%)	1065 (31.8)	647 (30.0)	418 (35.2)	0.002
Balloon Size for Post-dilatation, mm (mean ± SD)	14.6 ± 11.3	13.6 ± 11.2	16.3 ± 11.3	<0.001
Contrast Volume, mL (mean ± SD)	123.8 ± 69.3	121.4 ± 68.6	128.1 ± 70.3	0.009
Fluoroscopy Time, min (mean ± SD)	18.3 ± 12.5	18.2 ± 12.3	18.6 ± 13.0	0.357
Radiation Dose, Gy (mean ± SD)	11,619.7 ± 55,997.8	10,141.3 ± 49,365.5	14,638.0 ± 67,486.4	0.083
Closure Device Used, n (%)				0.014
Type 1	2617 (78.6)	1689 (78.4)	928 (78.9)	
Type 2	468 (14.0)	319 (14.8)	149 (12.7)	
Type 3	105 (3.2)	54 (2.5)	51 (4.3)	
Type 4	141 (4.2)	93 (4.3)	48 (4.1)	
Closure Device Failure, n (%)	152 (4.6)	107 (5.0)	45 (3.8)	0.144
Anesthesia (General), n (%)	223 (6.7)	150 (7.0)	73 (6.1)	0.380
Device Success, n (%)	3006 (89.7)	1943 (89.9)	1063 (89.3)	0.615

**Table 4:** Procedural complications and secondary outcomes stratified by gender in the study population (pre-PSM).

Parameter	Overall (n = 3353)	Female (n = 2162)	Male (n = 1191)	*p*-Value
Aorto-Iliac Dissection, n (%)	5 (0.1)	2 (0.1)	3 (0.3)	0.498
Annular Rupture, n (%)	6 (0.2)	5 (0.2)	1 (0.1)	0.590
Valve Recapture, n (%)	178 (7.4)	119 (7.5)	59 (7.1)	0.771
Valve Embolization, n (%)	39 (1.2)	27 (1.2)	12 (1.0)	0.649
Need for Second Valve, n (%)	54 (1.6)	31 (1.4)	23 (1.9)	0.340
Myocardial Infarction, n (%)	13 (0.4)	9 (0.4)	4 (0.3)	0.946
Emergency Surgery, n (%)	19 (0.6)	15 (0.7)	4 (0.3)	0.281
Coronary Obstruction, n (%)	5 (0.1)	4 (0.2)	1 (0.1)	0.797
Cardiac Tamponade, n (%)	34 (1.1)	30 (1.5)	4 (0.4)	0.008
Vascular Complications, n (%)	505 (15.1)	370 (17.1)	135 (11.3)	<0.001
Minor Vascular Complications, n (%)	305 (9.1)	213 (9.9)	92 (7.7)	0.046
Major Vascular Complications, n (%)	215 (6.4)	166 (7.7)	49 (4.1)	<0.001
Arrhythmias, n (%)				0.718
No Arrhythmias	2977 (97.7)	1938 (97.6)	1039 (97.8)	
1 Type	8 (0.3)	5 (0.3)	3 (0.3)	
2 Types	53 (1.7)	37 (1.9)	16 (1.5)	
3 Types	5 (0.2)	2 (0.1)	3 (0.3)	
4 Types	4 (0.1)	3 (0.2)	1 (0.1)	
Aortic Regurgitation, n (%)	69 (2.5)	50 (2.8)	19 (2.0)	0.225
Mean Post-procedural Gradient, mmHg (mean ± SD)	8.0 ± 4.5	8.2 ± 4.7	7.6 ± 4.1	<0.001
Post-procedural Aortic Regurgitation, n (%)	209 (6.3)	127 (6.0)	82 (7.0)	0.285
Post-procedural Ejection Fraction, % (mean ± SD)	58.8 ± 9.8	59.9 ± 8.9	57.0 ± 10.8	<0.001
New Left Bundle Branch Block (LBBB), n (%)	377 (12.4)	242 (12.2)	135 (12.7)	0.720
Persistent LBBB, n (%)	283 (17.9)	188 (18.3)	95 (17.3)	0.659
New Atrial Fibrillation, n (%)	143 (4.9)	103 (5.5)	40 (4.0)	0.099
New Pacemaker Implantation, n (%)	397 (11.8)	241 (11.1)	156 (13.1)	0.104
New Transient Ischemic Attack (TIA), n (%)	26 (0.8)	19 (0.9)	7 (0.6)	0.476
Minor Bleeding, n (%)	300 (9.7)	192 (9.6)	108 (10.0)	0.784
Major Bleeding, n (%)	139 (4.3)	106 (5.1)	33 (2.9)	0.004
Life-threatening Bleeding, n (%)	74 (2.3)	58 (2.8)	16 (1.4)	0.016
Blood Transfusion, n (%)	247 (7.4)	192 (8.9)	55 (4.6)	<0.001
Valve Thrombosis, n (%)	13 (0.4)	9 (0.4)	4 (0.3)	0.946
Acute Kidney Injury, n (%)	221 (7.5)	163 (8.5)	58 (5.7)	0.009
Hospitalization for Heart Failure Related to Valve Disease, n (%)	206 (8.8)	138 (8.9)	68 (8.5)	0.784
Endocarditis, n (%)	21 (1.0)	13 (0.9)	8 (1.0)	1.000
Reintervention Required, n (%)	8 (0.4)	4 (0.3)	4 (0.5)	0.666

**Table 5:** Clinical outcomes stratified by gender in the study population (pre-PSM).

Variable	Overall (n = 252)	Female (n = 170)	Male (n = 82)	*p*-Value
Outcome, n (%)	252 (7.5)	170 (7.9)	82 (6.9)	0.337
Death, n (%)	195 (5.8)	128 (5.9)	67 (5.6)	0.786
Stroke, n (%)	77 (2.3)	55 (2.5)	22 (1.8)	0.243
Cardiac death, n (%)	143 (4.3)	90 (4.2)	53 (4.5)	0.761
Procedural death, n (%)	15 (0.4)	12 (0.6)	3 (0.3)	0.323
In-hospital death, n (%)	42 (1.3)	35 (1.6)	7 (0.6)	0.016

**Table 6:** Baseline characteristics of the study population (post-PSM).

Parameter	Overall (n = 1688)	Female (n = 844)	Male (n = 844)	*p*-Value
Age, years (mean ± SD)	81.5 ± 5.9	81.5 ± 5.8	81.6 ± 6.1	0.723
Male, n (%)	844 (50.0)	0 (0.0)	844 (100.0)	<0.001
Height, cm (mean ± SD)	165.4 ± 8.4	161.6 ± 6.8	169.1 ± 8.1	<0.001
Weight, kg (mean ± SD)	73.5 ± 14.6	70.2 ± 14.7	76.8 ± 13.7	<0.001
Body Surface Area, m^2^ (mean ± SD)	1.8 ± 0.2	1.7 ± 0.2	1.9 ± 0.2	<0.001
Body Mass Index, kg/m^2^ (mean ± SD)	27.3 ± 5.1	27.3 ± 5.5	27.3 ± 4.6	0.733
Hypertension, n (%)	1444 (85.6)	718 (85.1)	726 (86.2)	0.545
Dyslipidemia, n (%)	680 (53.6)	330 (53.7)	350 (53.6)	1.000
Diabetes Mellitus, n (%)	512 (30.4)	254 (30.1)	258 (30.7)	0.836
Insulin Use, n (%)	165 (11.9)	76 (10.7)	89 (13.0)	0.211
History of Smoking, n (%)	176 (17.8)	63 (12.1)	113 (24.4)	<0.001
Chronic Obstructive Pulmonary Disease (COPD), n (%)	337 (20.0)	168 (19.9)	169 (20.1)	0.971
Pacemaker/ICD, n (%)	206 (12.2)	94 (11.1)	112 (13.3)	0.197
Atrial Fibrillation (AF), n (%)				0.078
No AF	1115 (66.3)	550 (65.3)	565 (67.3)	
Paroxysmal AF	542 (32.2)	285 (33.8)	257 (30.6)	
Persistent AF	1 (0.1)	0 (0.0)	1 (0.1)	
Long-standing Persistent AF	24 (1.4)	7 (0.8)	17 (2.0)	
Previous Cardiac Surgery, n (%)	251 (14.9)	117 (13.9)	134 (15.9)	0.265
Previous Myocardial Infarction (MI), n (%)	325 (19.3)	162 (19.2)	163 (19.4)	0.952
Previous Percutaneous Coronary Intervention (PCI), n (%)	507 (30.1)	249 (29.5)	258 (30.7)	0.636
Baseline Creatinine, mg/dL (mean ± SD)	1.2 ± 0.8	1.2 ± 0.9	1.3 ± 0.8	0.146
Estimated Glomerular Filtration Rate (eGFR), mL/min/1.73 m^2^ (mean ± SD)	54.3 ± 24.7	50.3 ± 23.5	58.4 ± 25.3	<0.001
Chronic Kidney Disease (CKD), n (%)	1072 (63.5)	593 (70.3)	479 (56.8)	<0.001
Dialysis, n (%)	41 (2.7)	18 (2.3)	23 (3.1)	0.459
Peripheral Arterial Disease, n (%)	225 (13.4)	117 (13.9)	108 (12.8)	0.586
Previous Stroke, n (%)	199 (11.8)	98 (11.6)	101 (12.0)	0.859
NYHA Class III/IV, n (%)	1126 (66.9)	562 (66.9)	564 (67.0)	1.000
Society of Thoracic Surgeons (STS) Risk Score, % (mean ± SD)	4.7 ± 3.4	4.8 ± 3.0	4.6 ± 3.7	0.373

Abbreviations: AF = atrial fibrillation; BSA = body surface area; COPD = chronic obstructive pulmonary disease; eGFR = estimated glomerular filtration rate; ICD = implantable cardioverter defibrillator; MI = myocardial infarction; NYHA = New York Heart Association; PCI = percutaneous coronary intervention; STS = Society of Thoracic Surgeons risk score.

**Table 7:** Pre-procedural imaging parameters stratified by gender in the study population (post-PSM).

Parameter	Overall (n = 1688)	Female (n = 844)	Male (n = 844)	*p*-Value
Mean Pressure Gradient, mmHg (mean ± SD)	43.2 (15.7)	42.9 (16.4)	43.4 (14.9)	0.485
Aortic Valve Area, cm^2^ (mean ± SD)	0.8 (3.5)	0.7 (0.3)	0.9 (4.9)	0.246
Severe Aortic Stenosis, n (%)	176 (20.3)	84 (20.3)	92 (20.3)	1.000
Ejection Fraction, % (mean ± SD)	56.3 (11.1)	56.2 (11.0)	56.5 (11.2)	0.634
Annulus Area, cm^2^ (mean ± SD)	10.0 (13.3)	9.7 (8.8)	10.2 (16.6)	0.422
Annulus Perimeter, mm (mean ± SD)	61.5 (23.2)	61.2 (23.3)	61.7 (23.1)	0.655
Aortic Angle, degrees (mean ± SD)	48.1 (9.9)	48.0 (10.1)	48.3 (9.8)	0.492
Aortic Angle > 49°, n (%)	771 (45.7)	380 (45.0)	391 (46.3)	0.625
Porcelain Aorta, n (%)	178 (10.6)	90 (10.7)	88 (10.5)	0.970
Aortic Valve Calcification (≥moderate), n (%)	770 (76.1)	401 (76.2)	369 (75.9)	0.967
LVOT Calcification (≥moderate), n (%)	299 (29.5)	164 (31.1)	135 (27.8)	0.273

Abbreviations: LVOT = left ventricular outflow tract.

**Table 8:** Procedural characteristics of the study population (post-PSM).

Variable	Overall (n = 1688)	Female (n = 844)	Male (n = 844)	*p*-Value
Transfemoral access, n (%)	745 (63.1)	366 (60.9)	379 (65.3)	0.128
Predilatation, n (%)	873 (51.7)	398 (47.2)	475 (56.3)	<0.001
Evolut R valve, n (%)	673 (39.9)	378 (44.8)	295 (35.0)	<0.001
Evolut PRO valve, n (%)	224 (13.3)	106 (12.6)	118 (14.0)	0.430
Evolut R/PRO valve, n (%)	897 (53.1)	484 (57.3)	413 (48.9)	0.001
ACURATE neo valve, n (%)	791 (46.9)	360 (42.7)	431 (51.1)	0.001
Valve size, n (%)				<0.001
23 mm	59 (3.5)	26 (3.1)	33 (3.9)	
25 mm	7 (0.4)	3 (0.4)	4 (0.5)	
26 mm	218 (12.9)	140 (16.6)	78 (9.3)	
27 mm	6 (0.4)	2 (0.2)	4 (0.5)	
29 mm	617 (36.6)	318 (37.7)	299 (35.5)	
Annulus size category, n (%)				<0.001
Large (L)	365 (21.6)	148 (17.5)	217 (25.7)	
Medium (M)	341 (20.2)	173 (20.5)	168 (19.9)	
Small (S)	74 (4.4)	34 (4.0)	40 (4.7)	
Annulus size in 3 categories, n (%)				0.010
1	123 (7.3)	51 (6.0)	72 (8.5)	
2	537 (31.8)	294 (34.8)	243 (28.8)	
3	1028 (60.9)	499 (59.1)	529 (62.7)	
Post-dilatation, n (%)	556 (33.0)	253 (30.0)	303 (36.0)	0.011
Balloon size for post-dilatation (mean ± SD)	15.8 ± 11.2	15.0 ± 11.4	16.5 ± 11.1	0.060
Contrast use (mean ± SD, mL)	127.6 ± 72.4	123.5 ± 72.5	131.7 ± 72.1	0.023
Fluoroscopy time (mean ± SD, min)	18.5 ± 12.1	18.4 ± 11.5	18.6 ± 12.6	0.727
Radiation dose (mean ± SD, mGy)	12,246.3 ± 58,427.7	9134.8 ± 45,399.6	16,003.6 ± 70,916.2	0.058
Closure device used, n (%)				0.195
Type 1	1319 (78.8)	674 (80.0)	645 (77.6)	
Type 2	221 (13.2)	111 (13.2)	110 (13.2)	
Type 3	64 (3.8)	24 (2.9)	40 (4.8)	
Type 4	69 (4.1)	33 (3.9)	36 (4.3)	
Closure device failure, n (%)	71 (4.3)	41 (5.0)	30 (3.6)	0.212
General anesthesia, n (%)	124 (7.4)	69 (8.2)	55 (6.5)	0.212
Device success, n (%)	1506 (89.2)	757 (89.7)	749 (88.7)	0.583

**Table 9:** Procedural complications and secondary outcomes stratified by gender in the study population (post-PSM).

Variable	Overall (n = 1688)	Female (n = 844)	Male (n = 844)	*p*-Value
Aorto-Iliac dissection = 1 (%)	3 (0.2)	1 (0.1)	2 (0.2)	1.000
Annular rupture = 1 (%)	2 (0.1)	1 (0.1)	1 (0.1)	1.000
Recapture = 1 (%)	91 (7.6)	58 (9.4)	33 (5.7)	0.022
Valve embolization = 1 (%)	20 (1.2)	11 (1.3)	9 (1.1)	0.822
Need for second valve = 1 (%)	30 (1.8)	15 (1.8)	15 (1.8)	1.000
Myocardial infarction = 1 (%)	6 (0.4)	2 (0.2)	4 (0.5)	0.683
Emergency surgery = 1 (%)	7 (0.4)	5 (0.6)	2 (0.2)	0.449
Coronary obstruction = 1 (%)	2 (0.1)	1 (0.1)	1 (0.1)	1.000
Cardiac tamponade = 1 (%)	13 (0.9)	9 (1.2)	4 (0.5)	0.290
Vascular complications = 1 (%)	234 (13.9)	138 (16.4)	96 (11.4)	0.004
Minor vascular complications = 1 (%)	151 (9.0)	86 (10.2)	65 (7.7)	0.089
Major vascular complications = 1 (%)	92 (5.5)	57 (6.8)	35 (4.1)	0.024
Arrhythmia (%)				0.493
0	1490 (97.9)	758 (97.9)	732 (97.9)	
1	4 (0.3)	2 (0.3)	2 (0.3)	
2	23 (1.5)	13 (1.7)	10 (1.3)	
3	3 (0.2)	0 (0.0)	3 (0.4)	
4	2 (0.1)	1 (0.1)	1 (0.1)	
Aortic regurgitation = 1 (%)	31 (2.3)	18 (2.6)	13 (1.9)	0.493
Mean gradient post (mean (SD))	7.6 (4.1)	7.5 (4.0)	7.6 (4.2)	0.451
Aortic regurgitation post = 1 (%)	121 (7.2)	57 (6.8)	64 (7.7)	0.571
Ejection fraction post (mean (SD))	57.8 (10.2)	57.9 (9.9)	57.7 (10.6)	0.709
New LBBB = 1 (%)	202 (13.3)	106 (13.7)	96 (12.8)	0.675
Post LBBB = 1 (%)	141 (17.5)	82 (20.0)	59 (14.9)	0.072
New AF = 1 (%)	72 (5.0)	39 (5.2)	33 (4.7)	0.756
New PM = 1 (%)	194 (11.5)	96 (11.4)	98 (11.6)	0.932
New TIA = 1 (%)	9 (0.6)	6 (0.7)	3 (0.4)	0.530
Minor bleeding = 1 (%)	150 (9.7)	72 (9.2)	78 (10.2)	0.529
Major bleeding = 1 (%)	60 (3.7)	38 (4.6)	22 (2.7)	0.059
Life-threatening bleeding = 1 (%)	34 (2.1)	22 (2.7)	12 (1.5)	0.135
Transfusion = 1 (%)	116 (6.9)	77 (9.1)	39 (4.6)	<0.001
Valve thrombosis = 1 (%)	3 (0.2)	2 (0.2)	1 (0.1)	1.000
AKI = 1 (%)	105 (7.2)	65 (8.7)	40 (5.6)	0.029
Sepsis = 1 (%)	13 (0.9)	8 (1.1)	5 (0.7)	0.652
HF hospitalizations = 1 (%)	95 (8.1)	53 (8.6)	42 (7.5)	0.551
Endocarditis = 1 (%)	5 (0.4)	3 (0.5)	2 (0.4)	1.000
Reintervention = 1 (%)	1 (0.1)	1 (0.2)	0 (0.0)	1.000

**Table 10:** Clinical outcomes stratified by gender in the study population (post-PSM).

Variable	Overall (n = 1688)	Female (n = 844)	Male (n = 844)	*p*-Value
Outcome = 1 (%)	130 (7.7)	79 (9.4)	51 (6.0)	0.014
Death = 1 (%)	175 (10.4)	95 (11.3)	80 (9.5)	0.264
Stroke = 1 (%)	34 (2.0)	24 (2.8)	10 (1.2)	0.024
Cardiac death = 1 (%)	75 (4.4)	39 (4.6)	36 (4.3)	0.813
Procedural death = 1 (%)	6 (0.4)	5 (0.6)	1 (0.1)	0.220
Hospital death = 1 (%)	25 (1.5)	16 (1.9)	9 (1.1)	0.227

## Data Availability

Data are unavailable due to privacy and ethical restrictions.

## Abbreviations

The following abbreviations are used in this manuscript:

BSA	Body Surface Area
BMI	Body Mass Index
COPD	Chronic Obstructive Pulmonary Disease
ICD	Implantable Cardioverter Defibrillator
PCI	Percutaneous Coronary Intervention
eGFR	Estimated Glomerular Filtration Rate
NYHA	New York Heart Association
STS	Society of Thoracic Surgeons
LVOT	Left Ventricular Outflow Tract
